# Assessment of a non-invasive high-throughput classifier for behaviours associated with sleep and wake in mice

**DOI:** 10.1186/1475-925X-7-14

**Published:** 2008-04-11

**Authors:** Kevin D Donohue, Dharshan C Medonza, Eli R Crane, Bruce F O'Hara

**Affiliations:** 1 Department of Electrical and Computer Engineering, University of Kentucky, Lexington, KY 40506, USA; 2 Department of Biology, University of Kentucky, Lexington, KY 40506, USA

## Abstract

This work presents a non-invasive high-throughput system for automatically detecting characteristic behaviours in mice over extended periods of time, useful for phenotyping experiments. The system classifies time intervals on the order of 2 to 4 seconds as corresponding to motions consistent with either active wake or inactivity associated with sleep. A single Polyvinylidine Difluoride (PVDF) sensor on the cage floor generates signals from motion resulting in pressure. This paper develops a linear classifier based on robust features extracted from normalized power spectra and autocorrelation functions, as well as novel features from the collapsed average (autocorrelation of complex spectrum), which characterize transient and periodic properties of the signal envelope. Performance is analyzed through an experiment comparing results from direct human observation and classification of the different behaviours with an automatic classifier used in conjunction with this system. Experimental results from over 28.5 hours of data from 4 mice indicate a 94% classification rate relative to the human observations. Examples of sequential classifications (2 second increments) over transition regions between sleep and wake behaviour are also presented to demonstrate robust performance to signal variation and explain performance limitations.

## Background

All mammals, and perhaps all animals, sleep [1]. Adult humans typically spend 5–10 hours a day in sleep, and yet, the basic functions of sleep are still unclear [2]. Recent estimates suggest that 50 to 70 million Americans experience either chronic or intermittent sleep related problems, and each year sleep disorders add billions to the national health care bill, and many more billions in accidents caused by sleepiness [3,4]. The most accepted technologies currently used in sleep analyses of mammals include Electroencephalographic (EEG) and Electromyographic (EMG) recordings [5,6]. While these technologies accurately discriminate between the sleep and wake states through semiautomatic scoring of the signal, the required preparations and analyses (surgery, recovery, signal scoring ...) limit its application in large-scale experiments often needed for genetic studies with rodents. In addition, for characterizing certain behavioural trends in larger groups, high accuracy on a small scale provided by EEG/EMG may not be required.

This work develops a classifier that automatically scores general behaviours related to sleep and wake that can be scaled up for analyzing large numbers of mice for several weeks. The system is based on detecting motion through a single Polyvinylidine Difluoride (PVDF) sensor on the cage floor. The system tracks external behaviours through pressure change on the cage floor (i.e. breathing, walking, grooming), and is not expected to replace EEG and EMG for detecting sleep and its stages. However, it is more sensitive to subtle movements than other activity monitors (i.e. wheel running or beam breaking) allowing the detection of many movements missed by other technologies. The intended application of this work focuses on large-scale phenotypying studies in genetically diverse mice in order to identify genes and gene alleles that influence sleep [7]. Typical studies are done over days or weeks, with or without manipulations, such as sleep deprivation periods. Differences in the monitored sleep-wake related behaviours between different strains can then be characterized with macro statistics related to average daily sleep and average wake activity over periods of hours. As a high-throughput system, it can also be used to determine strain differences for Quantitative Trait Loci (QTL) mapping or to screen mutant mice and find those with unusual behaviours for further testing.

This study considers the classification of inactivity associated with sleep versus wake, and does not assess sleep relative to evaluations performed through EEG measurements. Therefore, experimental assessment was based on visual observation of mouse behaviour, which cannot reliably detect sleep. While there is a high correlation between sleep and observed behaviours, such as inactivity with eyes closed, further studies with EEG measurements need to be conducted to make claims of actual sleep detection. This study focuses on collecting large amounts of data to assess overall system performance relative to behaviours often associated with sleep and wake. The large and varied data set provides assessments useful for development of the classification algorithm and signal processing methods, which are the main contributions of this paper. Further studies to confirm performance of this system relative to actual sleep would require EEG measurements, and are beyond the scope of this work.

The first work using PVDF sensors to monitor behavioural activity of rodents was reported by Megens el al [8]. For this application the sensors were used to detect changes in motor activity in response to different drugs and dosages. The primary signals of interest were from the rodent's feet striking the partitioned sections of multiple piezoelectric films distributed on the cage floor. The respiratory movements were filtered out for this application. In contrast, the work of Flores et al [9] used a (PVDF) sensor over the cage floor and tested a neural network classifier for sleep states to that of scored EEG signals. This work showed the critical behaviour for detecting sleep was the regular motions associated with breathing while the mouse was in a sleep posture. Feet in motion and shifting of weight for various activities, such as rearing, grooming, sniffing, etc. create transient spikes and random envelope variations in the PVDF signals useful for distinguishing wake state behaviours from a sleep-state mouse. The work of Flores et al demonstrated strong correlations between the mouse motions transduced by PVDF sensor to sleep and wake states.

A more extensive study between mouse breathing and sleep was performed by Friedman, et al [10], where EEG, EMG, and ECG recording were made simultaneously with a full body plethysmograph. The EEG and EMG were signals were correlated to the following behaviours of active wake, quite wake, NREM sleep, and REM sleep. A consistent periodic breathing pattern was shown for both sleep states, as well as for the quiet wake. The identified breathing patterns were incorporated directly in this work to develop robust classifiers to capture the behaviours associated with sleep.

A different approach to non-invasive monitoring of mice behaviour for sleep related research was described by Pack et al [11]. This system used digital video analysis and position information from infrared beam breaking to assess sleep and wake behaviours in mice. The method applied an assumption that inactivity greater than 40 seconds was sleep. This method achieved an average accuracy of 92% in classifying sleep and wake, suggesting a strong correlation between inactivity (sleep posture) and actual sleep.

The PVDF sensor system described by Flores et al overcame the limitations of having to connect special sensors to the animal for assessing general trends in sleep behaviour. On the limited data tested, the classifier achieved up to 95% classification rate relative to results from scored EEG measurement; however, the system had several limitations for high-throughput applications. The major contributions of this work include engineering developments to make the system useful for high-throughput systems. This includes designing a classifier that can be computed in a reasonable amount of time, and developing a robust feature set to maintain consistent performance over expected variations of the senor sensitivity and mice.

The first limitation of the system by Flores et al [9] was that several of the 9 features used were computationally expensive. In particular, a similarity measure was introduced requiring extensive comparisons of segments taken at 40 different lengths with 1260 segments taken from other time locations within an 8 second window. The processing of data from a single mouse for one day could take as long as 10 hours of computational time on a desktop PC. A second limitation of the system was that some of classifier features were dependent on the signal amplitude. While signal amplitude is well-correlated with mouse sleep and wake behaviours, amplitude is also affect by variations in mouse weight and contact with the cage floor (posture). The use of signal amplitude in the automatic classification statistics also requires strict settings and tedious calibrations for all amplifiers used for each sensor. The system also used a "signal predictability" feature, which was computed from forward and backward prediction error, which varied with the overall signal amplitude. To overcome this limitation, all features for the classifier of this paper were extracted from signals normalized over the analysis window. Therefore, the features of the classifier introduced in this paper are amplitude scale invariant and directly related to the waveform shape.

Finally, the previous classifier of Flores et al [9] used a neural network. While neural networks have the capabilities to learn complex patterns in the feature vectors (i.e. creating nonlinear decision boundaries), they require the estimation of a large number of weight values for the neuron connections computed iteratively on a training sequence. The set of classifier weights relate to all the interconnected neurons and give little insight on the impact of each feature. In addition, the training set did not include mice exhibiting the breathing variations described in [10]. Therefore, the classifier performance may change significantly requiring a retraining of the network. The work in this paper develops a simpler 5-weight linear classifier, where the features are selected to include the broader variations in breathing patterns. Each feature is related to general signal properties, such as envelope variation, frequency, and periodicity that can be directly modify or computed through other means. The presentation of these underlying signal dynamics provides information and understanding to enable easier adaptation for other applications or account for new behaviours.

The Methods Section of this paper describes the sensor system and the transforms used in the signal characterization and feature selection. The Results Section describes the experiment used to assess performance and presents classification rates for various parameter settings in the classifier. The Discussion Section explains the impact of classifier details and shows examples of sequential classifications near transitions and ambiguous regions relative to human observation to explain performance. Lastly, the Conclusion Section summarizes results and limitations of the system.

## Methods

### Instrumentation and Data Collection

**Figure 1:**
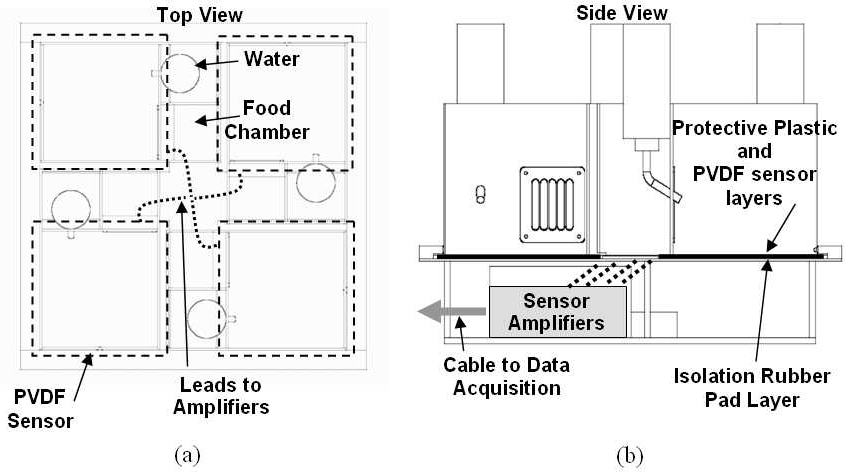
Quad cage and sensor system (a) top view showing cage walls on top of sensors on base (b) side view showing sensor layers on cage floor and connection to sensor amplifier.

A four-cage unit housed each mouse under test in individual compartments with sensors to capture pressure changes from anywhere on the cage floor. Figure 1 shows the top and side views of the cages with the sensing system. The cage was constructed from Lexan (polycarbonate). Four separate walled compartments with attached food/water structures with open bottoms were designed such that they can be inserted on the base and hold the sensor pad in place. Each PVDF sensing transducer covered the cage floor and extended 1.27 cm beyond the cage walls so the mice did not have access to the edges as shown in Fig. 1. The PVDF sensor was 17.78 cm by 17.78 cm square and consisted of a 110 μm thick dielectric, made by Measurement Specialties, Inc (Hampton, VA). Silver ink is sputtered on each side of the PVDF creating a conductive link from any position where pressure is applied. A protective plastic sheet (50.8 μm thick) was placed over the sensors to protect it from moisture and allow for easy cleaning. Additional bedding was placed on top of the plastic sheet for the animal's comfort. A 1.6 mm thick rubber pad made of Shore A 70 durometer silicon was placed between the sensor and the base to attenuate crosstalk from the pressure signals to other sensor pads. The side view in Fig. 1b shows the sensor placement with adjacent layers between the base and the cage walls. The chamber below the cage floors was 10 cm in height, and housed the instrumentation amplifiers for the sensors.

The capacitance of the PVDF sensor sheet is approximately 30 nF, and when coupled to the input differential amplifier, followed by a low-pass filter, effectively band-pass filtered the pressure signals with 3dB down points at 1.35 Hz and 20 Hz. The differential amplifier provides a high-pass effect and generates a linear gain of about 22. This stage was important for filtering out DC and low frequency noise/interference that could potentially push the amplifier into saturation for extended periods of time. Studies in sleep and breathing by Friedman et al [10] indicate rates as low as 2 Hz for NREM sleep in some genetic strains, and as high as 3.6 Hz for REM sleep in other strains. Therefore, the pass-band of the instrumentation amplifier filter was designed to cover this frequency range as well as significant harmonics. The amplified signals were then fed into a multi-channel data acquisition board (PCI 6224) and controlled with LabVIEW 7.1 software from National Instruments (Austin, TX). Data was sampled at 128 samples per second and quantized with 16 bits. For the performance analyses the signal classifications were implemented off-line with MATLAB 7.0 from the MathWorks, Inc (Natick, MA).

### Signal Transform and Critical Properties

**Figure 2:**
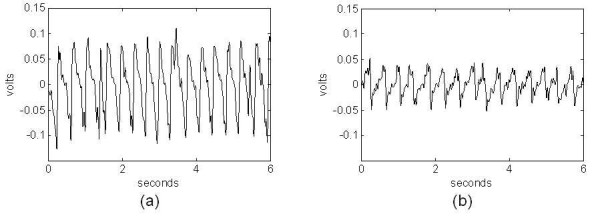
Example of piezoelectric signals corresponding to sleep from 2 different mice showing quasi-periodicity with (a) High-amplitude and (b) Low-amplitude.

**Figure 3:**
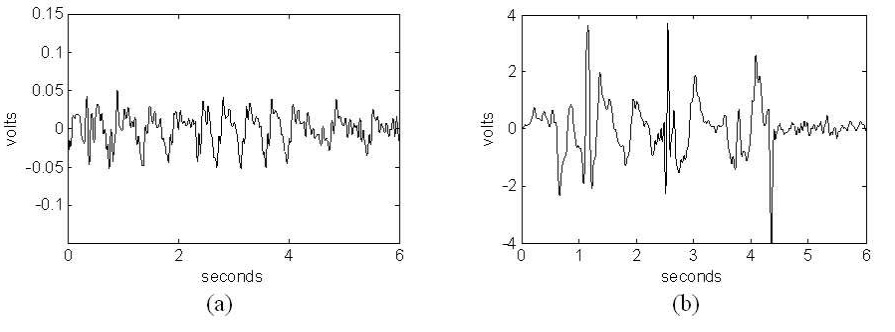
Examples of piezoelectric signals corresponding to wake from the same mouse showing random transient-like signals corresponding to (a) quite rest, and (b) motion across the cage floor.

Illustrative examples of sensor signals for different mouse behaviours are shown in Figs. 2 and 3. Figure 2 shows sleep behaviour signals for 2 recordings over separate sensors and amplifiers. The most obvious similarity is the quasi-periodic breathing signal (with periods between 0.3 and 0.4 seconds). The differences include amplitude/scale (due to a combination of the mouse size, sleep position, and amplifier gain) and shape of the periodic waveform. Examples of signals corresponding to wake behaviour are shown in Fig. 3. Figure 3a corresponds to a still mouse (quite wake) with eyes open. In this case the signal amplitudes are on the order of those of the sleep signal. This behaviour often precedes sleep, but typically differs in that the breathing pattern is not as regular as in the case of typical sleep signals with greater envelope variations. The frequency for quite rest often overlaps the breathing from sleep making it difficult to separate sleep from wake behaviours based on the pressure signals alone. However, other studies as well as our own observations indicate that mice are in this state only about 5% of the time [10]. Figure 3b corresponds to an active mouse moving across the cage (large amplitude spikes correspond to the feet striking the PVDF sensor). Signal characteristics corresponding to the typical wake behaviours can be described as random with strong transients (short-time, high amplitude). This is in contrast to the typical sleep behaviour patterns with consistent amplitudes and periodic variations.

Periodic behaviour can be characterized by the peaks in signal transforms, such as the autocorrelation (AC) function, and its frequency domain equivalent the power spectrum (PS) [12,13]. Transient and random signals can also be characterized by more involved analyses of these transforms. This work uses a variant of the PS, referred to as the collapsed average (CA), to more efficiently characterize transient behaviour as well as some forms of periodicity [14-16]. The CA is directly related to properties of the signal envelope and is computed by taking the AC of the positive frequencies of the spectrum.

**Figure 4:**
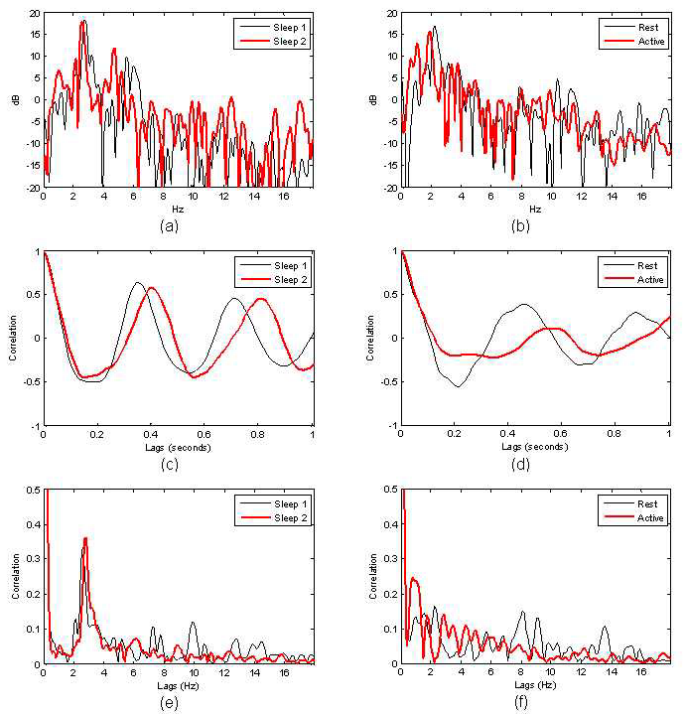
Examples of (a) PS for sleep signals of Fig. 1,(b) PS for wake signals of Fig. 2,(c) AC for sleep signals of Fig. 1,(d) AC for wake signals of Fig. 2,(e) CA for sleep signals of Fig. 1,(f) CA for wake signals of Fig. 2.

Examples of the PS for the sleep signals are shown in Fig. 4a and for wake signals in Fig. 4b. The most obvious differences for the 2 states are the higher peaks in the spectral region from 2 Hz to 4 Hz for the sleep signal. However, active and resting states also exhibit high peak values in or near this range, creating ambiguity and a need for additional features. Examples of the AC are shown in Fig. 4c for sleep and Fig. 4d for wake. Note the strong peak at a lag corresponding to the sleep period (in the neighbourhood of 0.3 and 0.4 seconds) in Fig. 4c that distinguishes it from the wake AC. The maximum peaks for wake also can occur in this region; however their magnitudes are typically smaller due to their lack of regularity over the analysis window.

Additional performance can be obtained by including features from the CA. Just as the AC detects periodic correlations in the time domain, the CA detects spectral redundancies due to dynamics of the envelope signal. Isolated transients are highly time-localized and result in linear phase spectral patterns. The CA for this pattern exhibits high amplitudes over broad frequency lag ranges. For any stationary random process, on the other hand, the expected value of the CA is zero for non-zero frequency lags [14-16]. Therefore, an increase in energy over a broad range of frequency lag values denotes the presence of non-stationarities result from motion activity (transients) over short intervals. Periodic behaviour with harmonic content, on the other hand, results in peaked CA values. The CA is therefore a good transform for separating the critical behaviours of this classifier.

An example of the CA is shown in Fig. 4e for sleep and Fig. 4f for wake. The key difference between sleep and wake is the distribution of energy over the frequency lags. The lags corresponding to the region from 2 Hz to 4 Hz show a relative increase over other lag regions for the sleep signals (expected frequency of breathing). For the wake state the response of the CA differs for active and quiet wake; however, in both cases low energy in the 2 Hz to 4 Hz region shows a reduction in the harmonic-rich periodicity characteristic of more regular signals. In the case of active wake, a relative increase in energy is observed for the lower lag values distributed over .5 to 2 Hz. This is typical of the CA response to isolated spikes. In the case of quiet wake, there is low energy everywhere except for frequencies below 0.4 Hz (the spectral region below 0.4 Hz was close to the effective resolution of the analysis window used to compute the CA and was not included in the analysis).

### Data Processing and Feature Extraction

Initial processing was performed to reduce noise and artefacts. Since the critical features for characterizing mouse motion were primarily in the spectral range of 0.5 Hz to 18 Hz, a band-pass FIR filter (Hamming windowed impulse response [12]) with order 511 was applied directly to the raw data. The filter removed low frequency noise.

Performance for the classifiers was examined for features computed with and without a logarithmic compression applied to the amplitudes. This limited the impact of large dynamic range variations on the feature estimation. The compressed signal segment was computed by:

$$\bar{x}[n]=\{\begin{array}{c} x[n]{\left(\frac{v[n]}{T_{M}}\right)}^{\left(1-\rho \right)}\text{ for }v[n]>T_{M}\\ x[n]\text{ for }v[n]\leq T_{M} \end{array}$$

where *v*[*n*] is the envelope of measured sensor signal *x*[*n*], *T*_*M *_is the compression threshold, and *ρ *is the compression factor. For the results of this paper, the envelope was computed from the processing window by taking the Hilbert transform magnitude of *x*[*n*], *T*_*M *_was taken as the median value of the envelope sample, and performance results were computed for *ρ *equal to 0.1 and also 1 (no compression).

For the PS computation $\bar{x}[n]$ is windowed by a Kaiser window [12]*w_β_*[*n*] with tapering parameter *β*. The Discrete Fourier Transform (DFT) of the windowed segment is taken to obtain:

$$\begin{array}{ccc} Y_{m}[k]=\sum_{n=0}^{N-1}w_{\beta }[n]\bar{x}\left[n-\frac{N}{2}+m\right]\exp\left(-\frac{j2\pi k}{N_{FFT}}\right) & \text{for} & 0\leq k<N_{fft} \end{array}$$

where *m *is the signal index for the sliding window over the data portion of interest, and *N*_*FFT *_is number of grid points for the spectrum evaluation (result of zero padding).

In order to make the features robust to changes in amplitude from factors not related to the sleep and wake behaviours, the normalized power spectrum used give by:

$$P_{m}[k]=\frac{{\left|Y_{m}[k]\right|}^{2}}{\sum_{k=0}^{N_{FFT}-1}{\left|Y_{m}[k]\right|}^{2}}$$

Examples of the power spectrum on the data were shown in Fig. 4a and 4b. For the spectrum computation, *N*_*FFT *_was taken as twice the segment length and rounded up to a power of 2, and the tapering parameter *β *was set to 6. The taper reduced the effective length of the segment to 34% of the extracted segment length. The effective length corresponds to intervals where window values exceeding 70% of the maximum window value (half-power points). For the examples presented in Figs. 4a and 4b, *N *was 512 (4 second intervals).

For the AC computation the data segment is processed as:

$$r_{m}[\lambda ]=\frac{1}{s_{m}}\sum_{n=0}^{N-1-\lambda }\bar{x}\left[n-\frac{N}{2}+m\right]\bar{x}\left[n-\frac{N}{2}+m+\lambda \right]$$

where *λ *is the sample lag, and *s*_*m *_is a normalization factor to the zero lag AC value equal to 1. Examples of the autocorrelation for sleep and wake signals are shown in Figs. 4c and 4d, computed over a 4 second interval for lags ranging from 0 to 1 second (128 samples).

The CA can be computed by:

$$C_{m}[\lambda ]=\frac{1}{S_{m}}\left|\sum_{k=1}^{N_{FFT}/2-\lambda }Y_{m}\left[k\right]Y_{m}^{*}\left[k+\lambda \right]\right|$$

where superscript * denotes the complex conjugate, and *S*_*m *_is a normalization factor to force the CA magnitude at 0 Hz to 1. The summation uses only half the spectrum (corresponding to the positive frequencies). For the CA to have the desired properties, only one-sided spectra are used. Care must be taken when interpreting the CA over the frequency lag axis. Artefacts are generated from the windowing and zero padding, which exist near the zero lag [14-16]. These areas in the CA are excluded from the analysis. Examples showing these properties are shown in Fig. 4e and 4f.

The classifier in this paper uses 5 features extracted from the functions computed in Eqs. (3)-(5). A more extensive analysis of over 30 features was performed in [17], which helped to identify the features analyzed here. The performance analysis in this work uses a more diverse and larger data set than that used in [17].

For both the AC and PS transforms the magnitudes and positions of all peaks were extracted. Specific ranges were determined for interpreting the peak location with respect to distinguishing sleep and wake behaviours. Since sleep typically results in breath motion signals between 2 Hz and 3.6 Hz [10], the spectral region from 1.5 to 4.5 Hz was designated as the sleep range for the PS. Strong peaks in this range suggest a high likelihood of sleep. Many of the features based on the PS typically did not result in good performance. This is due to impact of noise over the spectral range of interest (AC and CA significantly reduce the impact of stationary noise in the critical regions [16]). Only one feature of the set examined in [17] appeared to improve classifier performance by a few percentage points. This feature involved the maximum magnitude peak in the sleep range and global range (from 0.5 to 18 Hz), denoted by:

$$F_{1}(m)=10\log_{10}\left(\underset{n\in \text{Sleep}}{\max}\left({\hat{P}}_{m}[n]\right)\right)-10\log_{10}\left(\underset{n\in \text{Global}}{\max}\left({\hat{P}}_{m}[n]\right)\right)$$

where ${\hat{P}}_{m}$ denotes the peak magnitudes over the PS of Eq. (3), and the magnitude maximum is taken over the *n *indices corresponding to the sleep and global frequency ranges. If the dominate peak is in the sleep range, then *F*_1 _becomes 0. Greater negative values for *F*_1 _indicate a greater likelihood of wake behaviour. In the case when no peak is present in the sleep range, the value is set to the smallest value in the PS; however this condition rarely occurred.

The AC features were found to be the most powerful individual features when discriminating between sleep and wake states. When AC features are used independently, classifications agreement rates as high as 80% are achieved. Feature 2 is the AC peak magnitude in the sleep range given by:

$$F_{2}(m)=\left(\underset{n\in \text{Sleep}}{\max}\left({\hat{r}}_{m}[n]\right)\right)$$

where ${\hat{r}}_{m}$ denotes the peak magnitudes over the AC of Eq. (4). If no peaks exist in the sleep range, *F*_2 _is set to zero. Feature *F*_2 _includes the harmonic energy of a periodic waveform, unlike feature *F*_1_. Feature *F*_1_, however, includes the behaviour of other signal dynamics by including the global peak magnitude, thereby giving unique aspects of the signal characteristic. The second AC feature used is the distance of the maximum peak in the sleep range from the midpoint of the range (.34 seconds). This is denoted by:

$$F_{3}(m)=\left|{\hat{\tau }}_{m}-.34\right|$$

where ${\hat{\tau }}_{m}$ is the location of the AC peak in the sleep range (in seconds). If no peak is found in the sleep range, *F*_3 _is set to 0.34 (the largest possible value). This feature added minor improvement; however for some of the transitional and wake behaviours, such as those shown in Fig. 4d this peaks tends to shift away from the centre range (as well as reduce in amplitude).

Finally, the CA features provided additional information, especially for non-periodic signals, that enhanced performance when used with the AC features. The computation of the CA features involved summing up amplitudes over 2 critical frequency lag ranges as observed in Figs. 4e and 4f. The first range characterizes the transient signals typical of irregular motion given by:

$$F_{4}(m)=\frac{1}{\Delta_{T}}\sum_{\lambda \in \text{Transient}}C_{m}[\lambda ]$$

where the transient range includes frequency lags between 0.4 Hz and 2 Hz, and *Δ*_*T *_is a scaling factor corresponding to the summation range (1.6 Hz in this case). The last feature is computed by summing the CA over the range corresponding to sleep harmonics and is denoted by:

$$F_{5}(m)=\frac{1}{\Delta_{S}}\sum_{\lambda \in \text{Sleep}}C_{m}[\lambda ]$$

where the sleep range includes frequency lag indices corresponding to frequency values between 2 Hz and 4 Hz, and Δ_*S *_is the summation range (2 Hz in this case).

## Results

### Experiment Description

The classifier performance was tested against human observation of sleep and wake behaviour. Sleep studies typically require EEG signal scoring to correctly identify sleep [9]; however, this requires greater effort and more invasive apparatus, which limit the amount of data collected as well as limiting behaviours because of the invasive surgery. Human observation is sufficient to identify wake states when the mouse is doing some physical motion, but when the mouse is still with eyes open or closed, ambiguity can exist as to what the actual state is. Therefore, it can be expected that error will exist for the identification of sleep state by human observation. Most of which, however, will be in the form of missed arousals from sleep periods where eyes are closed.

An efficient interface was developed so the observer could simultaneously record a labelling voltage level with the sensor signals. This was implemented through a menu interface to a DC voltage generator with LabVIEW 7.1 software from National Instruments (Austin, TX). The observer selected a menu button on the computer terminal corresponding to the behaviour that sends a labelling voltage level to a channel on the data acquisition card collecting the motion signals. This resulted in a scored database, where signal segments corresponding to either sleep or wake behaviour could be automatically extracted for training or testing. The procedure circumvented the problem of hand scoring the signals, as done in [9], thereby enabling greater amounts of data with a greater variety of behaviours to be taken for training and testing.

The experiment involved four C57BL/6J mice, 2 males and 2 females, approximately six months of age. All experiments were performed in accordance with the protocols approved by the University of Kentucky Institutional Animal Care and Use Committee (IACUC). The four C57BL/6J mice used in these studies were obtained from The Jackson Laboratory (Bar Harbor, ME). The mice were six months of age at the time of study with the two males weighing 32–33 grams and the two females 24–25 grams. Both prior to and during the experiments, mice were on an approximately 12:12 Light:Dark cycle and were given food and water *ad libitum*.

The four C57BL/6J mice were placed in separate PVDF-sensor cages and 4 observer stations consisting of a laptop to control the function generator sending DC voltages levels to the PCI 6224 multi-channel data card. A total of 8 channels were used, 4 for the sensor signals and 4 for the labelling voltage levels coming from the observer stations. Three choices were available for the observer from a menu on the laptop screen, either "sleep," "wake," and "not certain." The sleep behaviour was identified as the mouse having closed eyes or head tucked under its body (eyes not observable), and remained still with possible intermittent stirring. If these conditions were not met, the wake behaviour was indicated. The mice were not observed continuously over the 3 day period, so in cases when no observations were made the "not certain" state was selected.

A total of 6 observers were used to result in 28.5 hours of observed sleep (10.5 hours) and wake (18 hours) behaviour over the 4 mice. It was noted that observers had a 2 to 4 second lag between the observed mouse state and the indication of the changed state. Therefore, data was only taken within labelled windows of constant behaviour greater than 10 seconds, and the 5 seconds before and after a transition were not used to account for reaction time in identifying the behaviour change. The result after censoring in this manner was 28.5 hours of labelled data.

From each segment in the training set a feature vector **f **was formed from features *F*_1 _through *F*_5 _(Eqs.(6) through (10)). The linear discriminate was computed from the training set using:

**v **= **Σ**^-1^(**m**_*s *_+ **m**_*w*_)

where **m**_*s *_and **m**_*w *_are the mean feature vectors computed over the sleep and wake segments respectively, and **Σ **is the mean of the covariance matrices computed from each class. The resulting weight vector **v **was applied to features vectors computed using the following decision rule:

$$\begin{array}{ccc} \text{if} & 0\leq f^{\text{T}}v, & \text{Decide Sleep}\\ \text{if} & 0>f^{\text{T}}v, & \text{Decide Wake} \end{array}$$

where superscript T denotes the transpose operation.

Data were parsed into segments for feature extraction and a bootstrap method randomly selected segments from each class to train and test the linear discriminate classifier. The bootstrap randomly selected 600 segments from each class to train, and 300 segments from each class to test (excluding those already chosen for the training). This was repeated 100 times for each classifier to compute the error percentages, from which the mean and standard deviation were computed. The standard deviations were converted to 95% confidence limits using the *t*-statistic and reported in the tables below along with the mean classification rate.

Classification rates were estimated for various signal processing parameter settings including the segment size and level of compression. The experiment was performed for different segment lengths (either 4s or 8s) and compression (either no compression or compression with *ρ *= 0.1). The amount of tapering from the Kaiser window was also considered; however, a significant difference did not result from changes over typical ranges (30% to 80% effective length reduction) so a tapering parameter corresponding to an effective segment length reduction of 34% was used and resulted in effective window lengths of 1.4s and 2.8s corresponding to the 4s and 8s windows, respectively. The use of short time intervals of scoring can be important for detecting brief changes in behaviour during sleep that may be an important for characterizing the activities of mammals [6], and are especially for mice [18].

### Classification Performance Results

Table 1 shows the classification percentages for agreement with human observation. The best rate being achieved was for the 8s segment using amplitude compression (94.3%). Comparisons between table entries show small but significant increases (greater than the 95% confidence limit range) for the larger window size and use of compression. Classification performance increases on the order of 0.6% to 2% for increased segment length, while a 2% to 3% increase results from the use of compression. The increased segment length results in greater spectral resolution, which improves the information contained in the spectral ranges. Preliminary investigations consider segment lengths near 1s for computing the PS and CA parameters. In these cases, however, the parameters made no effective contribution to classification performance, which dropped to about 70% as a result of using 1s segments. Therefore, for achieving performance in the 90% range, it is critical to have segment lengths at least 4s or greater.

**Table 1:** Percent classification agreement for combined behaviours

	Compression Factor	
Segment Length	ρ = 1 (no compression)	ρ = 0.1
4s	90.6 ± 0.5%	92.3 ± 0.4%
8s	91.2 ± 0.5%	94.3 ± 0.4%

Mean classification percentage for agreement between automatic and human observation classification for equal number of sleep and wake intervals with ± 95% confidence limits computed from bootstrap experiment. Results for variation in window length with and without amplitude compression.

Tables 2 and 3 show the agreement between human observation and the classifier for the separate cases of sleep and wake behaviour. Note the 2% to 6% better agreement rate for the wake segments over that for the sleep-only segment. The use of compression helps most dramatically with the sleep-only segments (resulting in 3% to 6% improvements), while for wake-only segments, the improvement is only about 1%.

**Table 2:** Percent classification agreement for sleep behaviour

	Compression Factor	
Segment Length	ρ = 1 (no compression)	ρ = 0.1
4s	87.8 ± 0.5%	90.1 ± 0.5%
8s	87.9 ± 0.5%	93.4 ± 0.5%

Mean classification percentage for agreement between automatic and human observation classification for only sleep intervals with ± 95% confidence limits computed from bootstrap experiment. Results for variation in window length with and without amplitude compression.

**Table 3:** Percent classification agreement for wake behaviour

	Compression Factor	
Segment Length	ρ = 1 (no compression)	ρ = 0.1
4s	93.5 ± 0.4%	94.4 ± 0.4%
8s	94.3 ± 0.4%	95.2 ± 0.4%

Mean classification percentage for agreement between automatic and human observation classification for only wake intervals with ± 95% confidence limits computed from bootstrap experiment. Results for variation in window length with and without amplitude compression.

In addition to the classification performance, the speed of the classifier was examined for applications to long periods with multi-channel recordings. In contrast to the previous neural network classifier [9], which required 10 hours of computation time per mouse per day, the processing for the features used for this classifier was performed in less than 6 minutes per mouse per day. For the system tested in this work, a 24-hour period of data for a single mouse requires less than 6 minutes of processing time, allowing for over 100 sensor channels to be processed and classified in real time (within 2 second intervals on a standard PC).

## Discussion

The results in the previous section show discrepancies between the automatic classifier and human observation on the order of 5 to 10%. To better understand the classifier performance and to examine the limitations of human observation, this section explains details of the classifier response to a variety of motion signals. The nature of the PVDF signals in regions where ambiguous and transitions states are also examined in light of the signal properties identified in earlier experiments [9].

All examples shown in this section used a segment length of 4s with a tapering window yielding an effective length of 2s, and this window was slid along the sensor signal in 2s increments. The classifier vector **v **was computed through bootstrapping experiments in the previous section where **v **from each randomly selected training set was averaged together (over the 100 trials). This resulted in the representative classification vector for feature weighting:

**v **= [0.0707, 2.9334, -3.0362, -1.2426, 0.8308]^T^

Each element of **v **corresponds to the weight for features *F*_1 _through *F*_5_. The negative signed-weights of Eq. (13) imply that large feature values for *F*_3 _and *F*_4 _imply a greater likelihood of wake. Feature *F*_3 _is the distance of the sleep peak from midpoint of the sleep range and *F*_4 _characterizes the dominance of transient spikes. In both these cases, larger values indicate greater likelihood of random motion or wake behaviour (especially *F*_4_). On the other hand, larger values for *F*_1_, *F*_2_, and *F*_5 _imply a greater likelihood of sleep, since these all relate to the dominance of the spectral energy and harmonic energy in the breath frequency range for sleep. So the general direction of the classifier weights computed from the training set is consistent with the observations of breathing and motion signals characteristic of sleep and wake behaviour observed in the experiments of [9,10].

**Figure 5:**
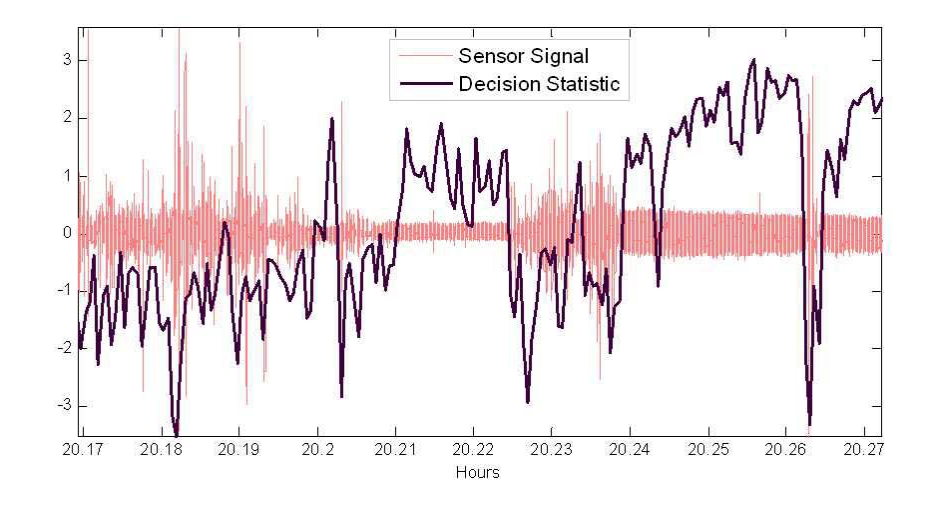
**Sensor signal with corresponding sleep-wake decision statistic computed every 2 seconds.** Long time-range to observe large scale signals behaviour over sleep and active periods with corresponding decision statistics

Figure 5 shows an 8 minute segment of the sensor signal and the corresponding sleep-wake statistics of Eq. (12). For this particular plot the *x*-axis denotes the hours into the experiment. Note the high concentration of large amplitude transient signal spiking before hour 20.21, as well as the burst between hours 20.22 and 20.24. This signal is characteristic of wake behaviour [9], and the classifier decision statistics respond properly with strong negative value in this range indicating wake.

The sensor signal in the period immediately after hour 20.21 shows smaller amplitudes compared to those after hour 20.24. The x-axis resolution is not sufficient to observe the signal periodicity in these intervals, but it is indeed present and similar to those in Fig. 2 and the ones described in [9,10]. Note from this plot that even for the same mouse, the signal amplitude changes for sleep. For the smaller amplitudes, however, the positive decision statistics is not as strongly positive as for the time range with the larger amplitudes. In this case the breathing signal is closer to the noise floor and features suffer more corruption of the random noise. Therefore, a potential source of error occurs if the breathing signal becomes too small relative to the noise floor. This can result from too much bedding between the mouse and the sensor floor, or setting the amplifier gain too low. Typically one would want to ensure that the sleep signal is not close to the noise floor before starting an experiment. The amplitude differences in the sleep signals in Fig. 5 resulted from the mouse repositioning itself after a short wake period. The larger amplitude range resulted from the mouse lying flat on the floor, and the smaller range resulted from the mouse tucking its head under its chest, and making less contact with the cage floor. However, in spite of these amplitude differences the computed features robustly classified the sleep and wake signals.

**Figure 6:**
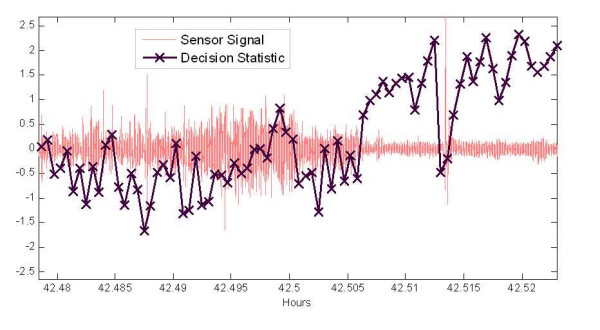
**Sensor signal with corresponding sleep-wake decision statistic computed every 2 seconds.** Long time-range to observe gross signal behaviour over sleep and active periods with corresponding decision statistics.

Figure 6 shows similar signals and statistics as those shown in Fig. 5 except over a shorter interval. The transition from rest (awake with no significant motion) to sleep occurs at about hour 42.506. There is a slight decrease in amplitude for the sleep state, but the main difference over the transition is the regularity (consistent amplitude and stronger periodicity) of the signal. As the breathing starts to become more regular in the quite active (or rest) state and subtle motion and body shifting decrease, the sleep-wake statistics become less negative and even positive for some epochs. This may be another source of discrepancy between the human observation and the classifier, as the observer would indicate the still mouse with eyes open as awake.

**Figure 7:**
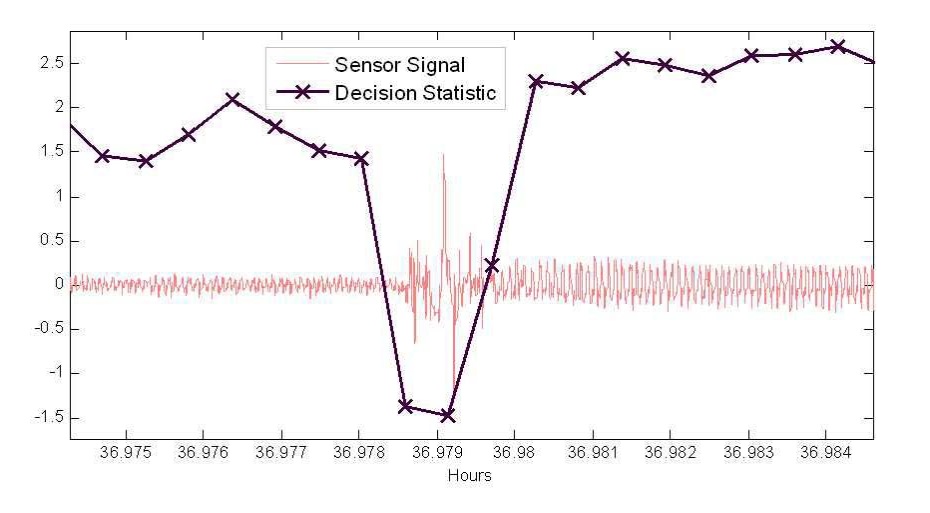
**Sensor signal with corresponding sleep-wake decision statistic computed every 2 seconds indicate by X markers.** Time series shows transition between rest and sleep and response of classifier decision statistics.

Figure 7 shows a case where an epoch of sleep was interrupted by a subtle motion during sleep as indicated by transient behaviour near the hour 36.979. In this case, the mouse shifted to change its sleep position, with the latter position resulting in better contact with the cage floor. In these cases the mouse typically keeps its eyes closed and the observer will not indicate this as a wake state, resulting in another source of discrepancy. However, as described in [6,18], mice exhibit many of short periods of wake during sleep resulting in a power-law distribution for contiguous sleep periods with a reported mean sleep bout of 5.9 minutes, while another study [18] with different criteria found a mean of 0.9 minutes in multiple different inbred strains of mice. Therefore, while this is a discrepancy between human observation and the classifier, it does not imply necessarily the classifier is incorrect. Further study and different methods will be needed to resolve how this transient should be classified.

Tables 2 and 3 show the agreement between human observation and the classifier is on the order of 2 to 5% better for the wake classifications than for the sleep. This is partly due to the fact that the wake states were typically less ambiguous in the observation (i.e. a moving mouse), and the non-detected transients via human observation cited in the last paragraph. As mentioned in the previous section the amplitude compression was used to mitigate the impact of the transient signals in the classifier. Which it did to some degree, but strong transients, as those shown in Figs 5 through 7, still resulted in wake decisions (with compression). Tables 2 shows that compression has a significant impact on the classification rates for sleep (where these isolated transients exist), while it had little impact on the agreement rate for classifying wake segments as shown in Table 3. Therefore, the weaker motion transients and other envelope irregularities of the breath signal during sleep result in a significant degradation of the extracted feature without compression.

The question of whether these transients actually correspond to sleeping or aroused will need to be answered with experiments that compare the PVDF sensor signals to scored EEG/EMG signals, since the signals identified in [9] did not include these cases. EEG/EMG studies are also necessary to determine if REM vs. non-REM sleep can be distinguished through the pressure signal measured in this system. While respiration becomes more variable during REM sleep [10,19], the respiratory frequency and general pattern are similar [10]. The system described here has the potential to detect these differences; however, the degree to which it can discriminate these patterns to any useful level is not clear.

The simplifications and the emphasis on a robust feature set (amplitude independent) allow this system to be used in phenotyping studies requiring high-throughput of animal behaviour characterizations. Even with the level of accuracy relative to human observation shown here, the system is valuable for a variety of sleep studies. Especially for macro behaviour characteristics such as average daily sleep, or changes in average sleep percentages over intervals of several hours [7]. The averaging over one hour periods can include 1800 sleep-wake behaviour decisions. Thus, statistics characterizing the macro behaviour will typically result in more reliable numbers because of the cancelling out of errors on the small interval (sleep-for-wake and wake-for-sleep errors).

## Conclusion

This paper presented a sensor and classification system that can be used for high-throughput systems to identify rodent behaviours associated with sleep and wake states. The agreement with human observation was evaluated over a large data set with a variety of behaviours and resulted in classification rates of 90% and higher. These classification rates are similar to more complicated classifiers [9] and those that used multiple modes for characterizing motion [11]. The technology presented in this paper is especially suited for generating statistics that characterize sleep behaviours for large numbers of mice over long periods of time. Adaptations of the system can also be applied to classifying other behaviours that result in characteristic changes in pressure patterns on the cage floor, which may be useful for the development and testing drugs [7].

The classification performance between eyes-closed inactivity and wake behaviours achieved a classification percentage as high as 95%. Such classification performance (even with the ambiguities introduce by the human observation) is sufficient to identify mice with outlying behaviours useful for phenotyping studies. With further testing this system could be of considerable value for the identification of sleep related genes that typically require large numbers of mice for genetic mapping and statistical analyses [7]. The system, however, should not be considered as a replacement of more traditional assessments of sleep and wake, such as EEG and EMG, especially where changes in sleep and wake over short intervals need to be assessed. This system directly measured changes in pressure on the cage floor (i.e. walking, grooming, breathing ...), and its success for any application is limited to the degree to which these measurements and related features consistently correlate with the behaviours of interest.

## Competing interests

The author(s) declare that they have no competing interests.

## Authors' contributions

BFO initiated the original concept of the system and targeted its application to sleep and genetic studies. He contributed to writing a signification part of the background, discussion, and conclusion. EC contributed engineering ideas in the design and modelling of the sensors, electronics, and physical cage. He contributed to writing the system descriptions in the methods section. DCM performed initial classification research dealing with feature selection and contributed to writing the background section and assembling most of the bibliography. KDD contributed to defining and resolving engineering issues associated with scaling the system up for high-throughput applications. He also designed the experiment for the performance analysis as well as interpreted results. All authors read and approved the final manuscript.
